# Data Fusion Applied to the Leader-Based Bat Algorithm to Improve the Localization of Mobile Robots

**DOI:** 10.3390/s25020403

**Published:** 2025-01-11

**Authors:** Wolmar Araujo-Neto, Leonardo Rocha Olivi, Daniel Khede Dourado Villa, Mário Sarcinelli-Filho

**Affiliations:** 1 Department of Electrical Engineering, Universidade Federal do Espírito Santo, Vitória 29075-910, ES, Brazil; wolmar.araujo@ufes.br (W.A.-N.); daniel.villa@ufes.br (D.K.D.V.); 2 Department of Electrical Energy, Universidade Federal de Juiz de Fora, Juiz de Fora 36036-900, MG, Brazil; leonardo.olivi@ufjf.edu.br

**Keywords:** robot localization, optimization, data fusion, autonomous mobile robots

## Abstract

The increasing demand for autonomous mobile robots in complex environments calls for efficient path-planning algorithms. Bio-inspired algorithms effectively address intricate optimization challenges, but their computational cost increases with the number of particles, which is great when implementing algorithms of high accuracy. To address such topics, this paper explores the application of the leader-based bat algorithm (LBBA), an enhancement of the traditional bat algorithm (BA). By dynamically incorporating robot orientation as a guiding factor in swarm distribution, LBBA improves mobile robot localization. A digital compass provides precise orientation feedback, promoting better particle distribution, thus reducing computational overhead. Experiments were conducted using a mobile robot in controlled environments containing obstacles distributed in diverse configurations. Comparative studies with leading algorithms, such as Manta Ray Foraging Optimization (MRFO) and Black Widow Optimization (BWO), highlighted the proposed algorithm’s ability to achieve greater path accuracy and faster convergence, even when using fewer particles. The algorithm consistently demonstrated robustness in bypassing local minima, a notable limitation of conventional bio-inspired approaches. Therefore, the proposed algorithm is a promising solution for real-time localization in resource-constrained environments, enhancing the accuracy and efficiency in the guidance of mobile robots, thus highlighting its potential for broader adoption in mobile robotics.

## 1. Introduction

Autonomous robots, particularly Unmanned Ground Vehicles (UGVs), have been increasingly used in recent years, generating new navigation and control challenges. Among them, achieving accurate localization remains a cornerstone of successful autonomous operations in various environments [1,2]. High-resolution localization data, typically provided by motion capture systems, are crucial information for controlling the robot’s movement and are unavailable in many situations. Cheaper solutions use other advanced sensing technologies, such as Laser Distance Detection and Measurement (LIDAR). However, the computational demands of processing great amounts of sensor data often exceed the capabilities of mobile robotic platforms, mainly concerning resource-constrained settings [3,4].

The Laboratory of Intelligent Autonomous Robots (LAB-AIR), Department of Electrical Engineering of the Federal University of Espírito Santo (UFES), Brazil, where this work was developed, has made significant strides in controlling mobile robots. The laboratory’s infrastructure, based on the OptiTrack motion capture system ( https://optitrack.com/applications/robotics/ (accessed on 3 January 2025)), supports precise experiments. However, the impossibility of using such systems in external environments underscores the need for alternative, cost-effective, and robust localization methods to obtain the position of the robot being controlled [5].

This paper addresses this challenge and proposes, designs, and validates a novel bio-inspired algorithm based on the leader-based bat algorithm (LBBA) [6] for mobile robot localization. The idea underlying such a proposal is to use sensory data fusion to enhance the accuracy and robustness of location estimates. As it is derived from a bio-inspired algorithm, the proposed algorithm capitalizes on the inherent strengths of bio-inspired optimization algorithms, specifically the bat algorithm’s ability to navigate in complex environments using echolocation-like mechanisms. By incorporating readings from a digital compass, the modified LBBA we propose optimizes the distribution of computational resources, thereby reducing the complexity of the localization task and improving operational efficiency in known environments.

The main contribution of the paper is exactly the proposal of this modified leader-based bat algorithm, incorporating leader dynamics to guide the swarm towards optimal solutions, effectively minimizing the localization error in complex environments. The effectiveness of the proposed approach is demonstrated through a series of experiments that validate the algorithm and explore its applicability in realistic scenarios. Such experiments aim to locate a ground robot while being controlled to navigate on a known map [4,5,7,8]. Therefore, this article contributes significantly to applied research in mobile robot localization, offering a viable alternative for robot localization during navigation control. As a secondary contribution, the proposed system opens new possibilities for autonomous exploration in outdoor environments, becoming useful in many applications.

To address such topics, this text continues with Section 2, which reviews related works and positions our contributions within the current state of the art in the field, followed by Section 3, which presents the problem formulation and the theoretical foundations of our approach. Thereafter, Section 4 outlines the experimental setup and the results obtained in some navigation experiments. It also compares the performance of our algorithm against some state-of-the-art methods. Finally, Section 5 summarizes the main findings and gives some possibilities for future research.

## 2. Related Works

The seminal book Probabilistic Robotics [9] provides a comprehensive foundation for probabilistic approaches in robotics, emphasizing robustness and accuracy in the face of uncertainty, making it an essential reference in the field. Building on this, Xin-She Yang proposed the bat algorithm (BA) [10,11]: a novel metaheuristic inspired by the echolocation behavior of bats, which integrates the strengths of existing techniques like particle swarm optimization and harmony search. Through detailed explanations of its formulation and implementation, Yang shows that the bat algorithm outperforms traditional methods, such as genetic algorithms and particle swarm optimization, making it a promising tool for solving complex optimization problems.

In addition, recent advancements have concentrated on combining multiple sensory inputs to improve localization accuracy. Techniques like Simultaneous Localization and Mapping (SLAM) [12,13] have become central to this field, enabling robots to create a map of an unknown environment while simultaneously tracking their position within that map. These methods are especially valuable in GPS-denied environments, such as indoor spaces or dense urban areas, where traditional GPS signals are often unreliable.

Bio-inspired algorithms based on Sequential Monte Carlo Optimization [14], commonly known as particle filters, are more robust than Kalman filters because they preserve the full posterior probability distribution of the problem, allowing them to accommodate multiple hypotheses and non-Gaussian distributions. Since robot localization frequently involves handling multiple hypotheses, bio-inspired algorithms are particularly well-suited for localization tasks. Specifically, the bat algorithm demonstrates fast convergence to solutions [11], and its variants, such as the leader-based bat algorithm (LBBA) [6], provide improved performance and enable data fusion capabilities.

Integrating data fusion techniques with bio-inspired algorithms represents a promising direction in robotics research. By combining the strengths of each approach, namely, robust statistical foundations and adaptive, nature-inspired mechanisms, researchers aim to develop localization systems that provide high accuracy, are computationally efficient, and are adaptable to environmental changes. This work goes further on these foundations, proposing an LBBA version incorporating digital compass data to optimize the localization process. This innovative approach, the main contribution of this work, reduces computational overhead and accelerates convergence by considering leader dynamics to guide the swarm toward the most probable solutions, thus enhancing accuracy.

## 3. Problem Formulation

The problem of mobile robot localization involves determining a robot’s precise position and orientation within a given environment. This is critical to ensure effective navigation and task execution in known and unknown settings. Indeed, the localization problem can be formally defined within the state estimation framework, where the goal is to estimate the robot’s state vector based on noisy sensor measurements.

This work focuses on the localization of a unicycle-type robot, also called a differential robot. Due to its simplicity and effectiveness in various applications, such a robot is likely the most common ground robot. The objective is to estimate the robot’s position using measurements from a LIDAR sensor and a digital compass.

### 3.1. Uncertainty and Sensor Fusion

Localization involves dealing with uncertainties, often of different natures, and with distinct probability density functions. These uncertainties affect all aspects of the experiments, from sensor measurements to the robot’s displacement when executing control laws. They also affect the environment with which the robot interacts, deviating it from its original path and changing the environmental configuration, considering static and dynamic obstacles. To address these uncertainties, we employ a probabilistic approach that models the robot’s state as a probability distribution. The key challenge is updating this distribution based on new sensor measurements. Specifically, we use a particle filter approach, where a set of particles represents the posterior distribution of the state, each one with an associated weight. These particles are propagated through the state space according to the robot’s motion model and updated using sensor measurements.

The state estimation process can be divided into prediction and update steps. The prediction step uses the robot’s motion model to propagate each particle to a new position, which corresponds to take

(1) $$x_{t+1}^{i}=f\left(x_{t}^{i},u_{t}\right)+w_{t}^{i},$$

where $x_{t}^{i}$ is the state of particle *i* at time *t*, $f(x_{t}^{i},u_{t})$ is the state transition function, $u_{t}$ represents the control inputs, and $w_{t}^{i}$ is the process noise for particle *i*, typically modeled as Gaussian with covariance Q due to the Central Limit Theorem (CLT) [15].

The update step uses sensor measurements to update the weights of each particle. The measurement model is given by

(2) $$z_{t}=h\left(x_{t}\right)+v_{t},$$

where $h(x_{t})$ is the measurement function, and $v_{t}$ is the measurement noise, typically Gaussian with covariance R, also due to the CLT.

Each particle has its weight updated based on the likelihood of the measurement in such a way that

(3) $$w_{t}^{i}=w_{t-1}^{i}\cdot p\left(z_{t}|x_{t}^{i}\right),$$

where $w_{t}^{i}$ is the weight of particle *i* at time *t*, and $p(z_{t}|x_{t}^{i})$ is the likelihood of the measurement $z_{t}$ given the state $x_{t}^{i}$ of the particle.

Typically, a Gaussian probability density function based on the sensor measurements models the likelihood, reflecting the discrepancy between the actual sensor reading and the predicted measurement from the particle’s state. The weight $w_{t}^{i}$ represents the degree of importance of particle *i*. A higher weight means that the sensor measurements from the particle’s state closely match the actual sensor measurements, suggesting that the particle’s state is similar to the state of the robot. Therefore, particles with higher weights are more likely to be in locations close to that of the robot within the environment.

To model the uncertainties in sensor measurements, we use standard probabilistic formulations. Error covariances model the uncertainties associated with the robot’s position and orientation. The likelihood function for sensor data fusion is defined as

(4) p(z|x)=𝒩(z;h(x),R),

where z is the measurement vector, h(x) is the measurement function relating the state x to the measurements z, and R is the sensor noise covariance matrix.

Updating of the prediction and correction steps of the particle filter happens as new measurements are received, adjusting the probability distribution of the robot’s state according to

(5) $$x_{t+1}∼\int p\left(x_{t+1}|x_{t}\right)p\left(x_{t}|z_{1:t}\right)dx_{t},$$

with

(6) $$p\left(x_{t+1}|z_{1:t+1}\right)\propto p\left(z_{t+1}|x_{t+1}\right)p\left(x_{t+1}|z_{1:t}\right),$$

where $p(x_{t+1}|x_{t})$ is the state transition probability, and $p(z_{t+1}|x_{t+1})$ is the measurement likelihood.

The weighted set of particles approximates the posterior distribution of the state $p(x_{t}|z_{1:t})$, which means that

(7) $$p\left(x_{t}|z_{1:t}\right)\approx \sum_{i=1}^{N}w_{t}^{i}\delta \left(x_{t}-x_{t}^{i}\right),$$

where *N* is the number of particles and δ is the Dirac delta function. While Gaussian distributions typically model the individual likelihoods $p(z_{t}|x_{t}^{i})$, due to the Central Limit Theorem, the combination of these likelihoods in the particle filter framework does not necessarily result in a Gaussian posterior. Instead, this approach approximates the true posterior distribution of the robot’s state, which can have any shape, thus capturing the full posterior and allowing for multiple hypotheses and non-Gaussian distributions.

Unlike Bayesian processes that often assume a Gaussian posterior, the final posterior distribution in Monte Carlo methods, such as the bat algorithm, is not necessarily Gaussian. One of the main advantages of using Monte Carlo-based methods is their ability to approximate the full posterior distribution, which can have arbitrary shapes and accommodate multiple hypotheses. This flexibility allows for a more accurate representation of the uncertainties and the robot state in complex, dynamic environments.

Resampling is performed to prevent particle depletion and ensure that particles with higher weights are more likely to be selected for the next iteration. This step is critical, aiming to maintain a diverse and representative set of particles, which helps to track the posterior distribution over time accurately.

### 3.2. Leader-Based Bat Algorithm (LBBA)

Bats’ echolocation behavior inspires the leader-based bat algorithm (LBBA). Virtual bats explore the search space to find the best solutions. Each bat represents a candidate solution for the robot’s pose. The algorithm iterates through a series of movements and updates to refine these solutions.

Key components of the LBBA include:

- Initialization: the algorithm starts with a population of bats, each one with a random initial position and velocity;
- Leader Selection: a subset of bats are designated as leaders based on their fitness values. Leaders influence the movements of other bats, guiding the swarm towards promising areas of the search space;
- Movement Update: bats update their positions and velocities based on the current best solutions and the influence of the leaders. The updating equations are
  (8a) $$f_{i}=f_{\min}+\left(f_{\max}-f_{\min}\right)\beta ,$$
  (8b) $$v_{i}^{t}=v_{i}^{t-1}+\left(x_{i}^{t-1}-x^{*}\right)f_{i},$$
  and
  (8c) $$x_{i}^{t}=x_{i}^{t-1}+v_{i}^{t},$$
  where $v_{i}^{t}$ and $x_{i}^{t}$ are the velocity and position of bat *i* at time *t*, $x^{*}$ is the current best solution, $f_{i}$ is the frequency factor, and β is a random vector drawn from a uniform distribution in [0,1];
- Evaluation: Each bat’s position is evaluated using a fitness function that measures the discrepancy between the estimated position and the actual sensor measurements;
- Termination: The algorithm iterates until a stopping criterion is met, such as a maximum number of iterations or a convergence threshold.

Integrating the LBBA with sensor fusion techniques enables the efficient combination of LIDAR and compass data, for instance, to improve localization accuracy and robustness. The focus of this work is precisely the fusion of data provided by the LIDAR sensor with the robot orientation provided by a digital compass, and the results shown in the following validate this proposal.

The LBBA is a non-parametric filter with a finite number of individuals, each corresponding to approximately one region in the search space. Non-parametric filters are well-adapted to represent complex multi-modal beliefs, such as in the case of mobile robot localization. Similar to other bio-inspired algorithms, the LBBA represents the a priori state of individuals using a set of random samples. This characteristic enables the simultaneous representation of multiple optimal localizations and allows the algorithm to explore a much more extensive space of distributions than Gaussian models [9].

The optimization process through the standard bat algorithm (BA) uses both the best global $g^{⋆}$ and the best individual $x^{⋆}$. The reason for employing $x^{⋆}$ is to increase diversity in the search for the globally optimal solution [16].

Bats can be territorial, especially regarding their resting places or shelters. This territoriality can vary significantly depending on the bat species. Some species tend to be more solitary and defend their territories against other bats, while others are more social and live in large colonies, showing less individual territorial behavior.

Fruit bats, for instance, may defend feeding territories rich in resources, such as fruit trees, against other bats to ensure a constant food supply. On the other hand, bats living in large colonies, like those inhabiting caves, may exhibit more cooperative behavior within their colonies, although still showing territoriality against bats from rival colonies or other species.

Territoriality in bats can also be observed in the defense of resting places against intruders, in competition for mates during the mating season, and in the protection of offspring. The methods used to defend territories can include vocalizations, aggressive physical behavior, and scent marking. Considering that the leadership proposal characterizes the LBBA, creating a metric to represent scent marking (the leader’s area of influence) on the map becomes crucial.

To address this issue, we established the relationship between the total area of the map and the area of interest that the actual robot would cover. The proposal consists of finding the number of mobile bases necessary to cover the entire map area where the localization mission will be carried out, considering that this is the part of the robot that would effectively contribute to covering the map.

Thus, the total area of the map was defined as 6 m^2^, in the laboratory tests reported in the following, as well as the area of interest that each robot covers, adopting the area of the P3-DX robot used in the experiments as 0.1519 m^2^. Then, the number of robots needed to cover the map was determined by dividing the total map area $A_{map}$ by the area of interest $A_{robot}$ that would be covered by the robotic structure, which means

(9) $$N_{leaders}=\frac{A_{map}}{A_{robot}}.$$

The result was rounded to the nearest integer greater than or equal to the calculated value. In the proposed test scenario, nine leaders would be necessary to achieve complete and uniform coverage of the map area. This parameter is not linked to the quantity of the bat population since this constitutes the operational sphere of swarm algorithms. Thus, it significantly contributes to improving the LBBA, considering that the number of bat leaders is a constant that seeks to be related to the bio-inspired characteristics of bats. It also reduces the number of parameters to be adjusted in a non-empirical manner.

Using multiple leaders encouraged the development of several colonies running concurrently. This approach aims to reduce the randomness in flight patterns while searching for the global optimum, making exploring the solution space more efficient.

Initially, the LBBA establishes the number of bats, *N*, used in the optimization process, following

(10) $$M=\{N\in N^{+}|\forall n_{i},\exists \left(x_{i},v_{i}\right),\;0<i<N\},$$

where *M* represents the set of individuals $n_{i}$, each one having two characterizing parameters: the position $x_{i}$ and the speed $v_{i}$.

Among the *N* bats, some are chosen as leaders during the algorithm execution. The number of leaders is defined according to the complexity of the environment. Bats fly randomly with a velocity $v_{i}$ at a certain position $x_{i}$, varying the loudness $A_{i}$ to search for prey and the frequency of their emitted pulses $r_{i}$ in a range of [0, 1] depending on the proximity of the target. The loudness is assumed to vary from a large positive $A_{o}$ to a minimum constant value $A_{min}$. For each position, it is necessary to compute an objective function $O(x_{i})$, given by

(11) $$O\left(x_{i}\right)=\frac{m_{i}-m}{m},$$

where the parameter $m_{i}$ represents the measurement performed by the simulated sensor of the *i*-th individual and *m* is the measurement performed by the real robot sensor. The bats with the best weights become the leaders. The algorithm dynamically updates the optimal value function for the *i*-th individual, $f_{i}$, as well as its velocity and position. The parameters $f_{\max}$ and $f_{\min}$ represent the maximum and minimum frequency of the pulse emission rate, respectively, which are predefined variables. The velocity and position vectors are generated as

(12) $$\left\{\begin{array}{c} f_{i}=f_{\min}+\left(f_{\max}-f_{\min}\right)\beta \\ v_{i}^{t}=v_{i}^{t-1}+\left(x_{i}^{t}-x_{*}\right)f_{i}\\ x_{i}^{t}=x_{i}^{t-1}+v_{i}^{t} \end{array}\right.$$

where the parameter β represents the change of the pulse rate.

The above equation works with the strategy that the closest leader of each colony influences the search for the best position. The distance equation of each bat to the leaders is given by

(13) $$dist=\sqrt{X^{2}+Y^{2}},$$

where

(14) $$\left\{\begin{array}{c} X=\mathrm{Leader}.x-\mathrm{Bat}.x\\ Y=\mathrm{Leader}.y-\mathrm{Bat}.y \end{array}\right.$$

### 3.3. The Proposed LBBA

**Figure 1:**
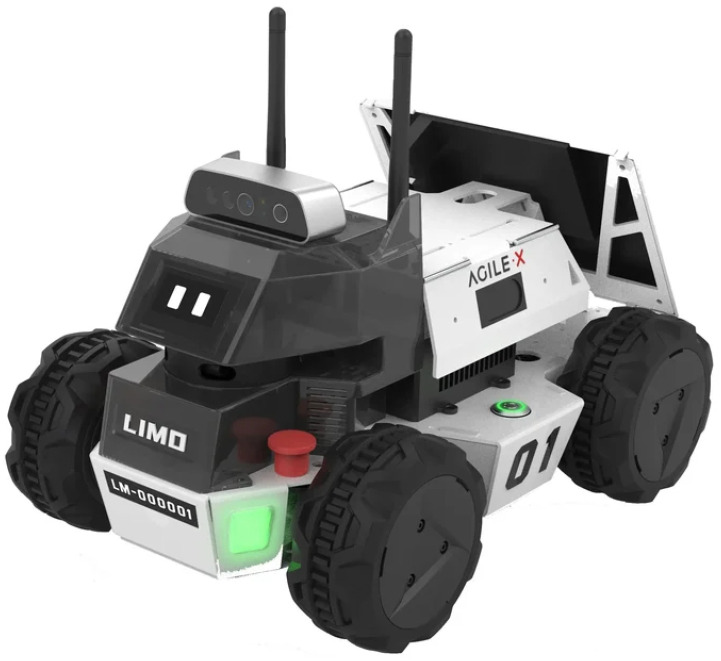
Robotic base used in the experiments run to validate the novel algorithm here proposed.

This study describes an experiment on the localization of mobile robots employing a differential robot that utilizes an RPLIDAR A2M12 sensor capable of measuring distances in $360^{\circ }$ at a high sampling rate. This allows for quick and efficient measurements, making it suitable for real-time applications like autonomous robot navigation. The robot used in the experiment was the AGILE X LIMO platform, configured as a differential one (see Figure 1), equipped with the LIDAR sensor.

The experiment employed foam mat tiles to simulate the characteristics of a mapped environment. These tiles enabled the creation of various test scenarios inside the laboratory, allowing significant versatility in configuring scenarios and making it easier to conduct experiments under different conditions.

One of the challenges faced was the high computational complexity resulting from the large volume of range measurements provided by the LIDAR sensor. To improve computational efficiency, the proposed localization algorithm was implemented in C++. A digital compass was also incorporated to provide precise information about the robot’s orientation on the map to improve localization accuracy. This integration allowed a more accurate determination of the robot’s orientation in the controlled environment. The fusion of LIDAR sensor data with the orientation information provided by the compass significantly improved the robot’s localization capabilities in complex scenarios. Moreover, this experiment offered valuable insights into the effectiveness of mobile robot localization in controlled environments, underscoring the importance of optimizing sensor fusion and sensing techniques to achieve accurate and efficient results.

**Figure 2:**
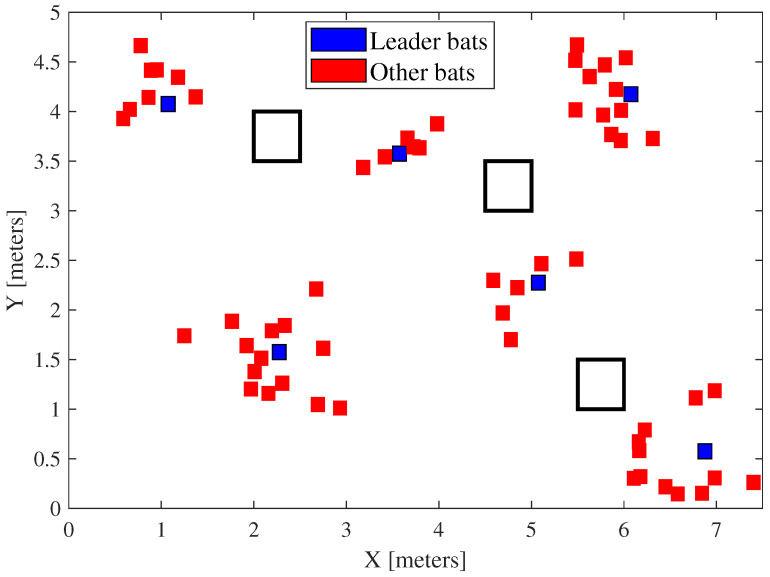
Diagram exemplifying the operation of the algorithm. The black squares are obstacles in the environment.

Figure 2 illustrates the basic foundation of the proposed LBBA. Initially, the bat population is randomly spread throughout the established limits. In this example, the blue bats are the leaders. At each interaction, the positions are updated according to the algorithm. This leads to an improvement in the direction of the target, as shown in the right part (first and second interactions). Notice that the leaders are in ambiguous positions or very close to the actual mobile robot position. This figure also shows that the population inside the region of interest (ROI) of the search space is instigated to follow the respective leader. This characteristic is extremely useful in complex environments and avoids “traps” in which the standard BA algorithm could enter.

**Figure 3:**
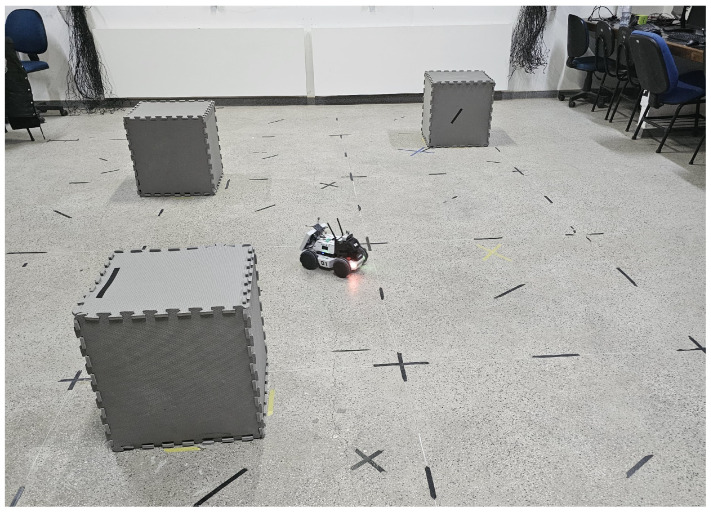
A snapshot exemplifying the robot navigating in a mapped environment.

Figure 3 reproduces the scenario within the Laboratory of Autonomous Intelligent Robots (Lab-AIR) facility, featuring the experiment with the LIMO robot navigating the mapped environment, mimicking the accomplishment of a mission inside a warehouse. The robot navigates around obstacles, including shelves, other robots, walls, and columns, some of which can be potentially hazardous. The robot autonomously traverses the known map, highlighting the importance of obtaining the most accurate position of the robot for trajectory control during the mission. However, wheel slippage, for example, can introduce uncertainties in the robot’s current position within the map. In such cases, one can provide accurate robot localization using the OptiTrack motion capture system, available in the Lab-AIR facility. However, this work aims to develop a localization strategy capable of determining the positions of the robot inside the laboratory in situations where a motion capture system is not available, a real possibility in most working environments.

Developing an efficient technique to locate the robot on a known map in a controlled environment is extremely important for research. For instance, it becomes necessary when GPS signals are unavailable or imprecise. Furthermore, the increasing use of drones for inspection and indoor monitoring highlights the relevance of this development [17]. Some proposals, such as that of [17], bring a practical approach to trajectory control, demonstrating that the model can be applied to complex trajectories. However, the main limitation lies in the dependence on a processing rate that requires specific hardware. Effectively, in natural and large-scale environments, this need can represent a significant challenge when adopting this solution.

This work aims to add an orientation sensor capable of providing the robot’s orientation within a known map and reducing the number of calculations required in the optimization algorithm. Thus, the proposed algorithm allows greater precision and speed in estimating the robot’s position on the map, whether for developing a SLAM in constructing a map or pursuing a trajectory, such as a lemniscate one.

**Figure 4:**
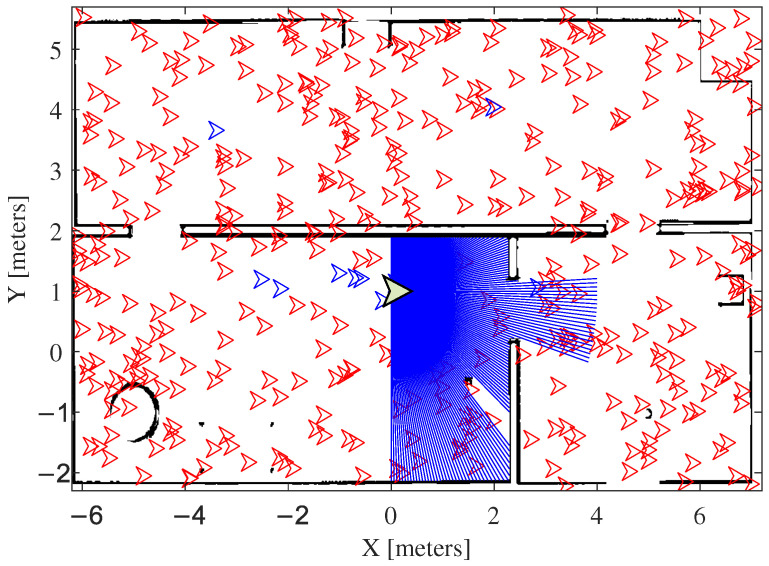
Particles distributed on the map, oriented according to a compass emulated by a motion capture system. The leader bats are in blue.

Our proposal adopts the robot’s orientation to direct the particles in the distribution process, which demands a sensor, a compass, for instance, to provide such information. However, the robot adopted in the experiments discussed here does not have a compass to provide information about its orientation. Then, the OptiTrack installation available in the test arena is used to obtain the robot orientation. Since the robot’s orientation is known, the particles are distributed according to this orientation, which limits particle dispersion, as illustrated in Figure 4.

**Figure 5:**
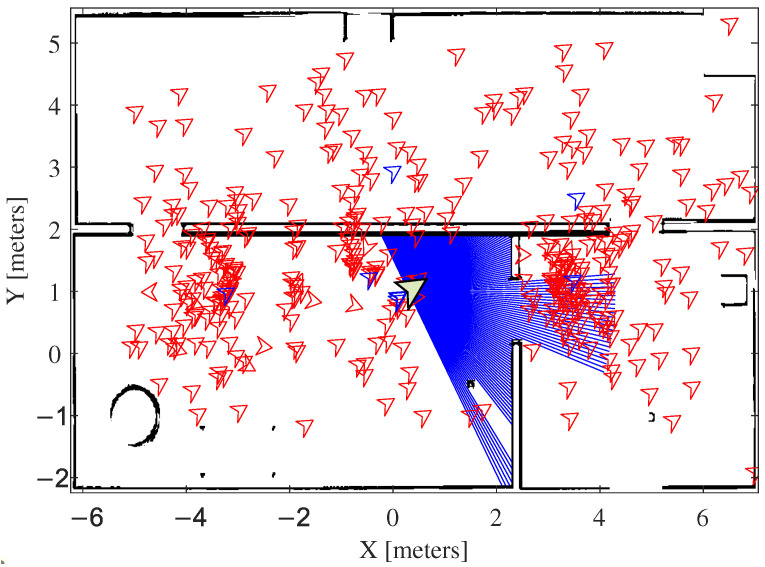
Particles distributed on the map, oriented according to a compass emulated by a motion capture system, after a slight motion of the robot. Once more, the leader bats are blue. Notice the grouping of the bats around the leaders.

As the robot moves, the simulated agents, simulating the bats’ behavior, must follow this motion, synchronized in linear and angular velocity. The digital compass plays an essential role in orientation, helping to estimate the bats’ positions within the known map. The simulation shown in Figure 5 demonstrates that, after an incremental movement of the robot, the bats begin their “flight” toward the leaders, highlighting the interaction and alignment with the movements of the leader bat.

**Figure 6:**
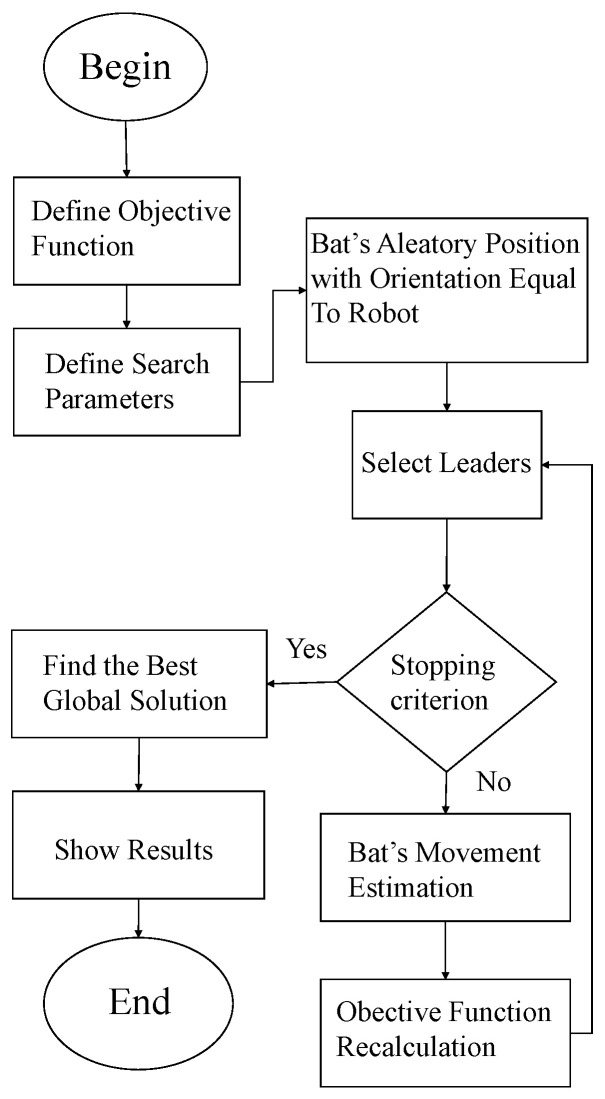
Flowchart corresponding to the proposed LBBA.

The flowchart of Figure 6 makes clear the central idea of the proposed enhanced LBBA. Initially, the algorithm defines the objective function and the search parameters. Then, the particles are scattered on the map, oriented according to the robot orientation, as illustrated in Figure 4. Upon achieving the objective function or the maximum number of iterations, the stopping criterion, the algorithm has found the global solution. Otherwise, it recalculates the objective function, repeating this recalculation until fulfilling the objective function or reaching the maximum number of iterations.

To further emphasize the importance of the digital compass, the proposed LBBA uses the orientation of the robot it provides to reduce the number of particles needed for accurate localization. The compass precisely estimates the robot’s orientation, allowing the algorithm to disregard particles with significantly deviating orientations. This reduction in the search space enhances the efficiency and speed of the localization process and maintains a high level of accuracy by focusing on the most probable positions. Thus, the digital compass is pivotal in optimizing the LBBA, enabling it to operate with fewer computational resources while delivering robust and precise localization. Therefore, the digital compass is crucial for the LBBA method proposed here because it significantly reduces the number of particles (bats) required for accurate localization. With the orientation provided by the compass, the algorithm can limit the dispersion of particles, focusing only on the most probable orientations. This results in the following:

- Reduction of Search Space: it limits the possible orientations of the robot, allowing for a more efficient and faster search;
- Improved Accuracy: the precise orientation from the compass helps better align the localization estimates with reality, increasing the algorithm’s accuracy;
- Reduction in Computational Complexity: the need for fewer particles reduces the computational load and allows real-time implementation of the algorithm.

## 4. Results

The experiments and simulations presented in this section used different scenarios to find the best configuration for locating a mobile robot in a controlled environment. The experiments used cameras of the OptiTrack system to provide a ground-truth reference, enabling the analysis of the effectiveness of the LBBA and providing reliable information for the robot’s (LIMO) orientation, emulating a digital compass.

### 4.1. Performance Associated to the Real Tests

This section describes the experiments conducted with the LIMO robot in a controlled environment, utilizing the proposed LBBA. The main objectives were to validate the accuracy of the global localization algorithm and evaluate the effectiveness of trajectory-tracking control in autonomous navigation tasks. The robot was equipped with a LiDAR laser sensor capable of performing 180 measurements per scan. In addition, the OptiTrack installation was used as a ground truth reference to validate the localization and trajectory-tracking control. Additionally, a digital compass mimicked by the OptiTrack system was incorporated into the system, helping to reduce the randomness in the algorithm’s estimates and guiding the particles toward a more accurate orientation, as mentioned before.

**Figure 7:**
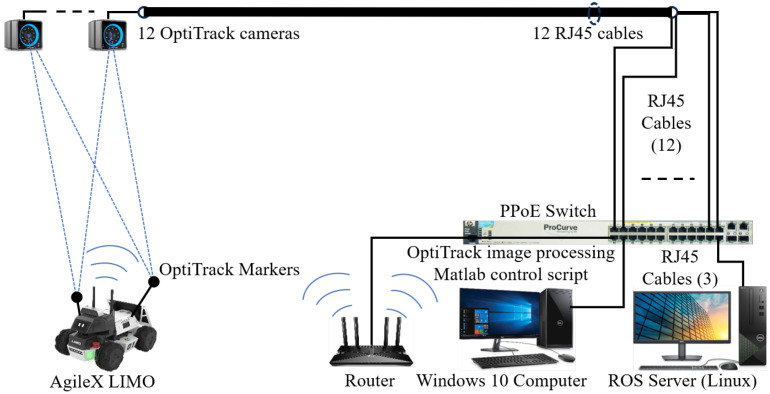
A sketch of the test arena where the experiments were run.

The experiments adopted a previously known map containing obstacles that avoid excessive ambiguities. Initially, the robot had no information about its exact location in the environment. The LBBA was used to perform global localization, and the tests demonstrated its effectiveness in quickly determining the robot’s initial position. This step is critical, as the mission only begins when the global localization returns a reliable position estimate. Figure 7 describes the test arena, consisting of a Linux PC running ROS *melodic*, which establishes Wi-Fi communication between the control computer and the robot, with the control signals sent to the vehicles at a rate of 30 Hz. As for the controller adopted, it was the kinematic one discussed in [18].

The mission began after the global localization phase, and during it, we applied the local localization algorithm. This algorithm focuses on a probability region along the robot’s movement trajectory, optimizing the use of particles in the area where the robot is. This procedure allowed the robot to maintain a suitable frequency of position updates for trajectory control during the missions.

**Figure 8:**
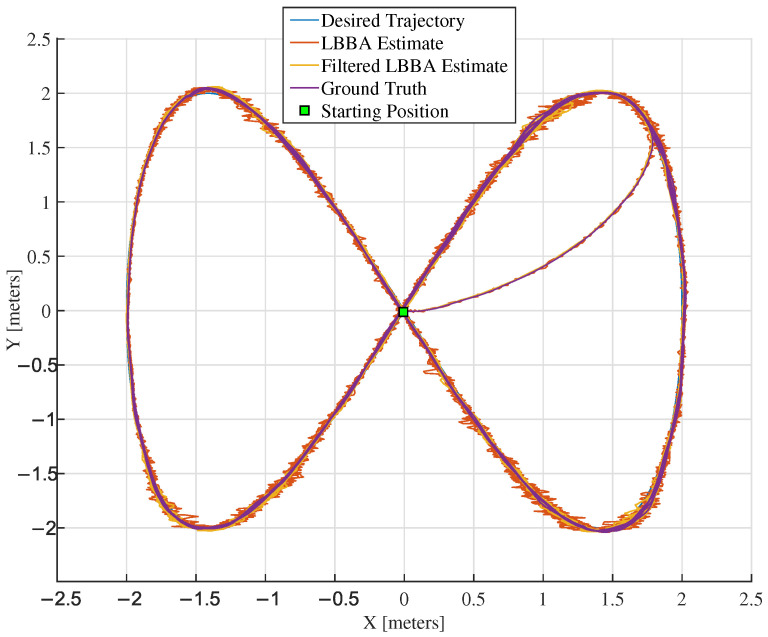
Lemniscate trajectory tracked and the algorithm efficiency obtained.

**Figure 9:**
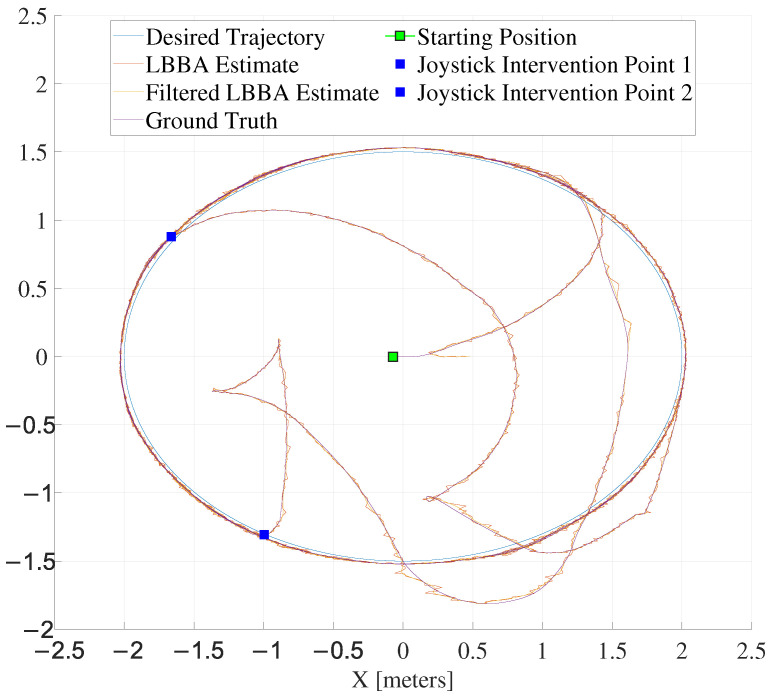
Elliptical trajectory tracked and the algorithm efficiency obtained.

The experiments used two trajectories: a lemniscate of Bernoulli and an ellipse. The lemniscate, shaped as an 8, is widely used in experiments on robot motion control to test the ability to smoothly and precisely control the robot movements because the continuous changes in direction and curvature make it challenging enough to validate control algorithms. Figure 8 and Figure 9 illustrate both trajectory-tracking experiments, respectively. In the second case, the ellipsoidal trajectory, human intervention through a joystick takes the robot to a point entirely out of the trajectory in two different moments along the navigation channel, marked as blue squares inside black boxes. As one can see from Figure 9, the localization system could provide the necessary information for the controller to guide the robot to resume the trajectory.

**Figure 10:**
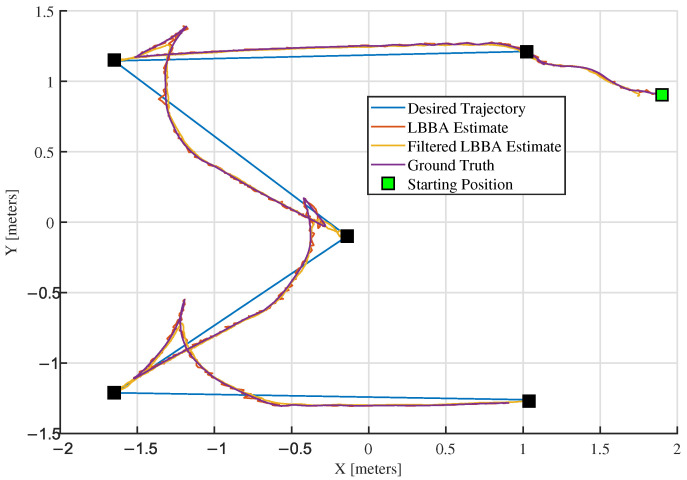
Position estimation for the experiment of waypoint navigation. The black squares are the points to be reached by the robot.

In addition to the two trajectory-tracking tasks, the robot also performed a positioning mission, programmed to reach five waypoints distributed throughout the map, as shown in Figure 10.

**Figure 11:**
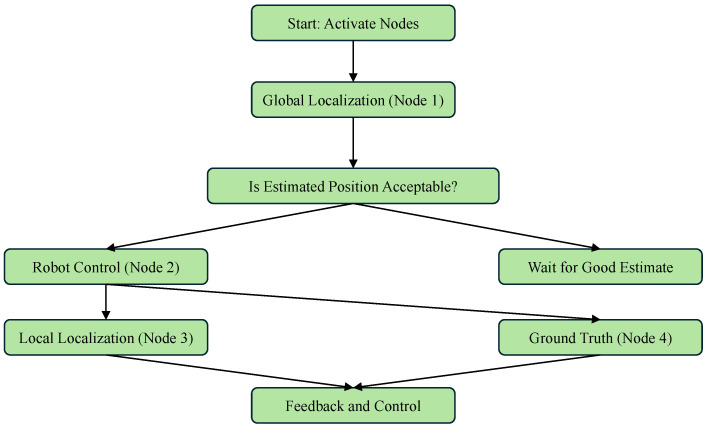
Flowchart of the localization algorithm based on random search (LBBA) implemented in C++.

Using the LBBA for position estimation was crucial for trajectory control and positioning success. Instead of spreading particles across the entire map, the local localization strategy allowed the use of a smaller number of particles without compromising the accuracy of the estimates. Figure 11 shows the flowchart of the localization algorithm, detailing the global and local localization steps.

The flowchart presented describes a robotic navigation system using a Robot Operating System (ROS), comprising several nodes responsible for the location and control of the robot. The process begins with the activation of all nodes: Global Location (Node 1), Local Location (Node 3), Robot Control (Node 2), and Ground Truth (Node 4). Initially, the Global Location node estimates the robot’s position in the entire map. If the estimated position is acceptable, the robot starts moving toward the desired trajectory/position, with the Robot Control node managing this movement. From this moment on, Local Location becomes responsible for continuously estimating the robot’s position during navigation. The Ground Truth node (based on the OptiTrack motion capture system) provides the robot’s orientation to the localization system and the real robot position for comparison with the one estimated by our algorithm. It also assists in correcting the robot’s orientation within the working environment. This feedback loop ensures accurate navigation and continuous adjustments as the robot moves across the map.

The close correspondence between the estimated and real trajectories demonstrated the algorithm’s accuracy, as shown in Figure 8 and Figure 9, which present an overlay of the trajectories estimated by the LBBA proposed here and the real trajectories provided by the OptiTrack system, considering the trajectory-tracking of a lemniscate of Bernoulli and an ellipse, respectively. There, one can see that the trajectory generated using the positions estimated by our algorithm is very close to the ground truth, which demonstrates the accuracy of the estimates.

Tools such as error graphs and boxplots illustrate the distribution of errors and evaluate the performance of the localization system. The estimates tend to converge to the reference values over time or during the algorithm’s execution, as evidenced by Figure 8 and Figure 9. Indeed, a visual analysis of overlapping line graphs allows the progressive approximation of the estimates to the ground truth to be verified. In the experiment related to the lemniscate trajectory, the localization algorithm demonstrated the ability to estimate the position accurately and efficiently over 15 min.

On the other hand, in the experiment in which the robot followed an elliptical trajectory, besides proving the ability of the proposed algorithm to follow the desired path, we also evaluated its robustness. A human operator using a joystick made interventions, forcing the robot away from the desired trajectory. The system, however, demonstrated to be able, after resuming the control of the robot, to resume the desired trajectory, validating the robustness of the proposed algorithm.

**Figure 12:**
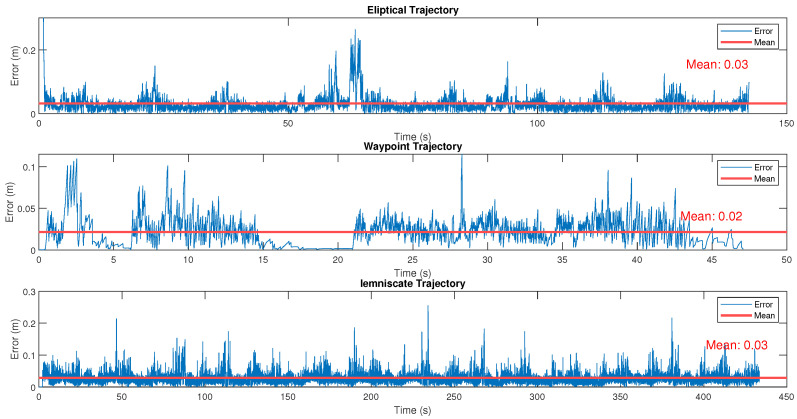
Euclidean errors over time for an elliptical trajectory, a waypoint sequence, and a lemniscate trajectory.

The accuracy of mobile robot position estimation is a crucial factor directly impacting performance in tasks related to online trajectory planning, motion control, and environmental interaction. This study performs a quantitative analysis of the position estimation error of the LIMO robot in three different types of trajectories: elliptical, waypoints, and lemniscate. The results are presented in the form of error graphs over time, providing a critical assessment of the robustness of the navigation system and the challenges encountered in each type of trajectory (Figure 12).

The analysis was performed by comparing the position the robot’s navigation system estimated with the real position obtained by high-precision sensors. The errors were recorded over time, and the results were visualized in graphs with a trend line representing the average error. This approach allowed a detailed analysis of the system’s accuracy in different navigation contexts.

The elliptical trajectory, characterized by continuous and smooth curves, presented a moderate variation in error over time. The maximum error recorded was approximately 0.2 m, with more pronounced peaks observed around 50 s (top graph). These peaks can be attributed to the system’s difficulty in maintaining high accuracy on curves, where slight deviations in the estimation of the angular orientation propagate to the linear position error. The average error on this trajectory was 0.03 m, showcasing the good performance of the estimation algorithm.

The waypoint navigation presented a distinct error behavior, with more pronounced fluctuations in the first 15 s. The maximum error recorded was approximately 0.1 m, with higher peaks between 30 and 35 s, which coincide with transition moments between different waypoints (center graph). This suggests the navigation system faces challenges during the robot’s reorientation at these transition points. The average error along this navigation channel was 0.02 m, the lowest among the three experiments analyzed, indicating that the robot could perform accurate position corrections throughout most of the route.

With its figure-of-eight pattern, the lemniscate trajectory introduced greater complexity due to the continuous variation in curvature and direction. The maximum error recorded was similar to that of the elliptical trajectory, around 0.2 m, but presented more frequent fluctuations over time (lower graph). The average error was 0.03 m, which reflects the ability of the navigation system to maintain an acceptable overall accuracy, even on a trajectory with continuous orientation changes.

The results show that the performance of the system in estimating the robot’s position varies according to the trajectory’s complexity. Trajectories involving continuous changes in curvature, such as the elliptical and lemniscate, presented higher error peaks, suggesting that the estimation and control algorithm are more sensitive to abrupt variations in the angular orientation and linear velocity. This behavior emphasizes the importance of information fusion with the compass sensor, especially when dealing with more complex trajectories, such as those analyzed here.

As Table 1 and Table 2 show, the filtered algorithm presents a lower error than a version without filtering. Therefore, using filtered data provided better conditions for controlling the robot during the missions. This behavior can be justified by the ability of the filters to smooth the sensory data, removing rapid oscillations and noise, which could induce undesired behaviors in the controller. The conclusion is that data smoothing allows the controller to generate more consistent and stable movement commands, resulting in more efficient robot behavior. Filtering also reduces the controller’s sensitivity to small fluctuations, which, in the case of raw data, could result in abrupt and ineffective corrections, highlighting the advantage of using filtered data to improve control performance.

The Root Mean Square Error (RMSE) index is the basis of such an analysis. It measures the difference between the estimated and real values (ground truth) over time and is a metric widely used to evaluate the accuracy of localization algorithms. Such an index is calculated as

(15) $$\mathrm{RMSE}=\sqrt{\frac{1}{n}\sum_{i=1}^{n}{\left({\mathrm{Estimate}}_{i}-\mathrm{Ground}\;{\mathrm{Truth}}_{i}\right)}^{2}}$$

where *n* is the number of samples, ${\mathrm{Estimate}}_{i}$ are the estimated values, and $\mathrm{Ground}\;{\mathrm{Truth}}_{i}$ are the true values. Besides presenting lower RMSE values, the filtered data provided smoother and more predictable data, which resulted in improved robot control.

Additionally, filters help mitigate temporary error spikes that can severely impact robot performance if not properly corrected. By providing a more stable and predictable data set, the controller can predict the robot’s movement more effectively, facilitating smoother tracking of complex trajectories, such as lemniscate and elliptical. In this way, the filtered data provide greater overall stability to the control system, allowing the robot to maintain robust and more efficient performance throughout the mission.

**Figure 13:**
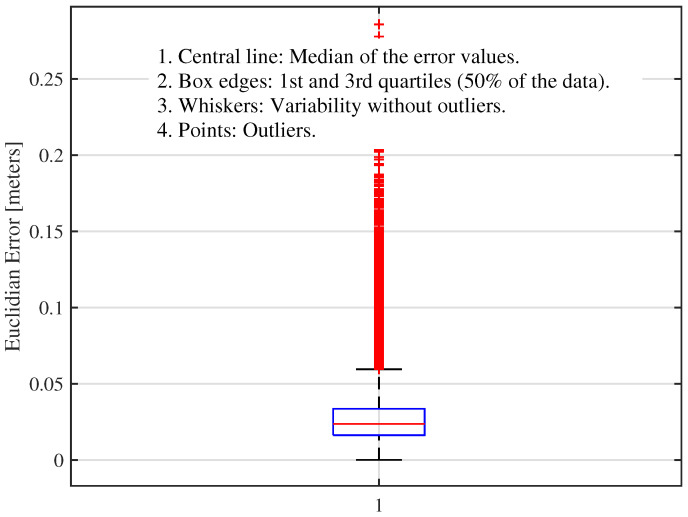
Boxplot of the Euclidean error distribution in the lemniscate trajectory experiment (estimate vs. ground truth).

Figure 13 shows the analysis of the errors of the localization algorithm in a lemniscate trajectory experiment, comparing the estimates with the real position of the robot. The distribution of errors, illustrated by the boxplot, demonstrates a significant concentration of values close to zero, as the interquartile range indicates. The median of the error values, represented by the red line inside the box, is slightly below the center, suggesting a slight asymmetry in the data. The narrow width of the interquartile range indicates that most of the errors remain within a narrow range, reflecting the algorithm’s consistency most of the time. However, many outliers are observed above the box, revealing more significant errors at specific moments. These atypical values indicate that, although the algorithm’s overall performance is accurate, there are situations in which the robot’s localization error increases considerably. Furthermore, the whiskers, which show the variability of errors without considering outliers, are relatively short. This reinforces that most errors remain within a small range, with occasional exceptions that indicate deviations in the system’s behavior at certain moments of the experiment.

**Figure 14:**
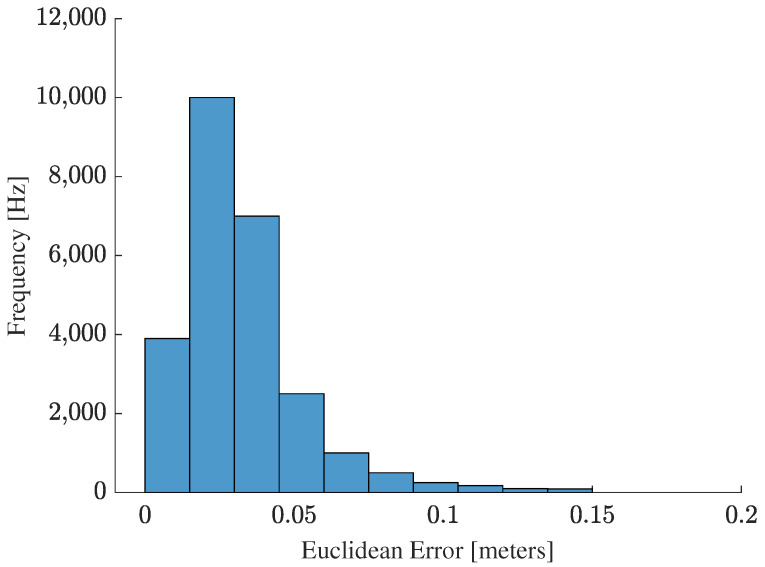
Histogram of the distribution of Euclidean error along the lemniscate trajectory.

The histogram of Figure 14 shows the distribution of the Euclidean error along the lemniscate trajectory. Analysis of this graph reveals that most of the errors are concentrated in low values, with the highest frequency of occurrences around errors close to zero, which indicates the excellent overall performance of the localization algorithm.

A sharp drop in frequency is observed as the error increases, with a few values above 0.05, suggesting that most of the algorithm’s estimates are pretty accurate. However, there are a small number of more significant errors, reaching values around 0.15 or more, which indicates the occurrence of some estimates with more significant errors but which are rare.

The greater concentration around small errors, combined with the low frequency of more significant errors, reinforces the idea that the algorithm performs well in most cases, with a few exceptions where the error is more pronounced. This asymmetric distribution, with a long tail on the right, suggests that the most significant deviations are sporadic and may be related to the specific conditions of the experiment, such as noise or temporary limitations in the localization process. Therefore, the histogram confirms that the localization algorithm has a high accuracy most of the time, with few moments of significant error.

**Figure 15:**
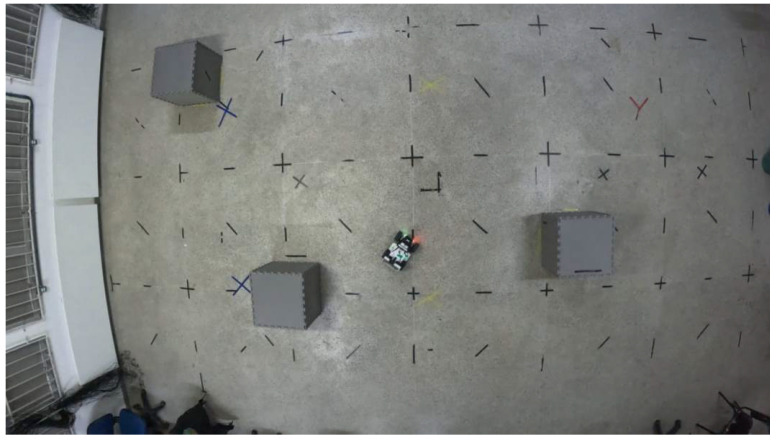
Top view of real tests with LIMO.

Therefore, one can conclude that all the experiments were successfully conducted, demonstrating that the LBBA proposed here is effective for localization and trajectory control in known environments, establishing itself as a promising tool for applications in autonomous robotics. Finally, Figure 15 shows the LIMO robot in action during the experimental tests to give an idea of the experimental setup.

### 4.2. Performance Evaluation Through Simulated Tests

In this section, we compare the performance of the leader-based bat algorithm (LBBA) as proposed here against the performances of two other bio-inspired algorithms: the Manta Ray Foraging Optimization (MRFO) algorithm and the Black Widow Optimization (BWO) algorithm, also focused on optimization and also applicable to mobile robot localization. These three algorithms are based on natural behaviors and are designed to handle complex global optimization problems in search spaces with multiple local optima. This makes them particularly suitable for the robotic localization problem. Although MRFO and BWO yielded strong results, our LBBA proposal outperformed them in accuracy and robustness, indicating that it may offer distinct advantages in this context.

Essentially, LBBA is an extension of the bat algorithm (BA). The difference, as detailed in this text, is the inclusion of multiple leaders that direct the swarm to specific regions of the search space. This feature provides a unique distributed exploration framework, efficiently covering larger areas and avoiding traps in local optima. Considering robotic localization, the algorithm can obtain more accurate estimates of the robot’s initial position by exploring different regions simultaneously and adjusting itself to avoid suboptimal solutions that could result in significant errors. By its turn, MRFO, inspired by the foraging behavior of manta rays, incorporates three distinct strategies, namely chain, cyclone, and somersault, which seek a balance between exploration and intensive exploration in promising regions. Each strategy uses structured moves to ensure that the algorithm can switch between a global search and localized exploration, adapting to the configuration of the search space. This makes MRFO suitable for situations where adaptation to new regions is critical. However, this approach may require more particles to maintain accuracy in restricted areas, such as a region of interest (ROI) [19]. The BWO, in turn, is based on the reproductive and cannibalistic behavior of the black widow spider, where low-fitness individuals are eliminated to optimize exploration. This process of discarding unpromising hypotheses improves the algorithm’s convergence rate, which helps reduce the initial calculation time in complex environments [20]. However, the dynamics of cannibalism, by rapidly reducing particle diversity, can limit its adaptability in restricted areas, such as an ROI, making it difficult to obtain an accurate robot position.

Accurate and rapid robot localization in a mapped environment initially requires a global estimate of its position. For this task, particles are randomly distributed throughout the map, allowing the algorithm to compare the readings of a real sensor with simulated readings to identify the robot’s position. However, this initial phase requires a significant amount of particles, which increases the computational cost. At this point, the robustness of LBBA in exploring multiple regions with independent leadership proves advantageous, ensuring broad coverage and rapid convergence to an accurate initial estimate.

After obtaining a reliable initial position, the algorithm switches to a local localization procedure based on the definition of an ROI, a circle centered on the last estimated robot position, with a radius of 20 cm, inside which the robot is supposed to be. This refinement significantly reduces the number of particles required since the algorithm focuses the search on the area with the highest probability of containing the robot. This improves computational efficiency, allowing for real-time precise localization without requiring the entire map to be explored again.

A relevant aspect contributing to the accuracy and robustness of localization is the integration of a compass, which assists in orientation and avoids unfavorable positioning hypotheses. Data fusion between the compass and other sensors (LIDAR and odometry) reduces variability and the need for more particles, improving the algorithm’s reliability and enabling localization with a lower computational cost. This feature is especially useful in ROI, where orientation is more sensitive to deviations.

### 4.3. Algorithm Performance

With its multi-leader structure, the LBBA proposed here is highly effective in both the global localization and ROI phases. Its ability to quickly explore large regions and focus on a specific region allows for high accuracy with fewer particles throughout the robot’s navigation. As proposed here, integrating a compass and using multiple leaders ensure additional robustness, minimizing calculation time without compromising accuracy.

While effective in the global localization phase, the MRFO requires more particles in the ROI phase to maintain good accuracy. This is because its cyclone-based and somersault-based approaches, while excellent for initial exploration, may not be as adaptable in the ROI, where more localized and less cyclical movements are preferable for continuous and accurate robot localization.

The BWO algorithm has proven fast in global localization by eliminating low-fitness particles, making an efficient initial convergence easier. However, as the robot moves through the ROI, the reduction in diversity imposed by cannibalism limits its ability to adapt to slight variations, which can compromise accuracy in areas with multiple potential solutions nearby.

Considering the characteristics of these three algorithms, we now compare them based on the following three criteria:

1. **Convergence Speed in Number of Epochs**: The number of epochs required for each algorithm to converge to the final solution, that is, the estimated *x*, *y* robot position and orientation θ. This criterion is important for assessing how quickly the algorithm reaches a viable solution, especially relevant for real-time applications in embedded computer resources;
2. **Final Solution Quality**: Calculating the standard deviation of the difference between the algorithm’s estimated position and the robot’s true position allows for assessing the solution quality. The standard deviation reflects the precision level of the algorithm’s estimate, with lower values indicating a closer approximation to the robot’s true position. This metric is particularly crucial in autonomous navigation systems, where high precision is necessary to avoid undesirable deviations;
3. **Computational Time**: We evaluate the computational efficiency by measuring the execution time of the algorithm in seconds. The same hardware was adopted in each case to allow a fair comparison. This criterion is essential as it indicates the computational cost of each algorithm, a metric especially relevant in systems with limited processing resources.

### 4.4. Comparison Results and Discussion

**Figure 16:**
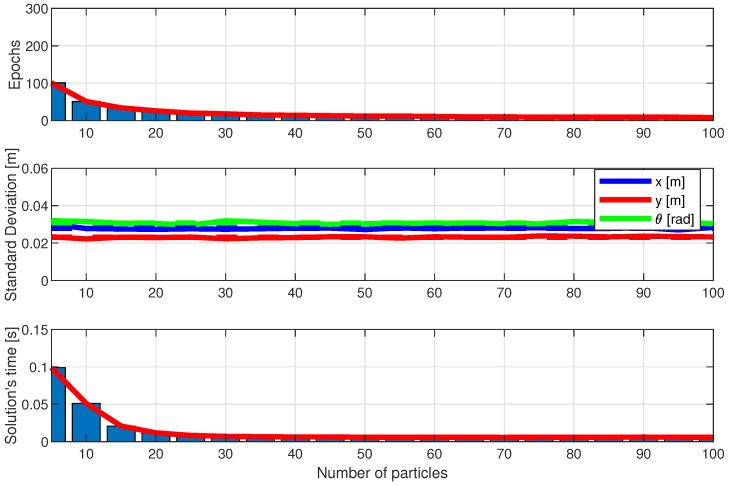
Performance of the LBBA proposed here in terms of the number of particles: convergence speed (in epochs), error standard deviation (in meters), and computational speed to solution (in seconds).

**Figure 17:**
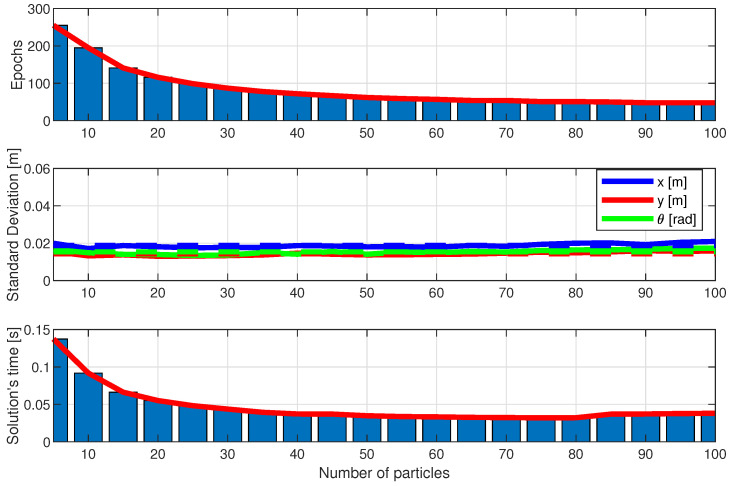
Performance of the BWO algorithm in terms of the number of particles: convergence speed (in epochs), error standard deviation (in meters), and computational speed to solution (in seconds).

**Figure 18:**
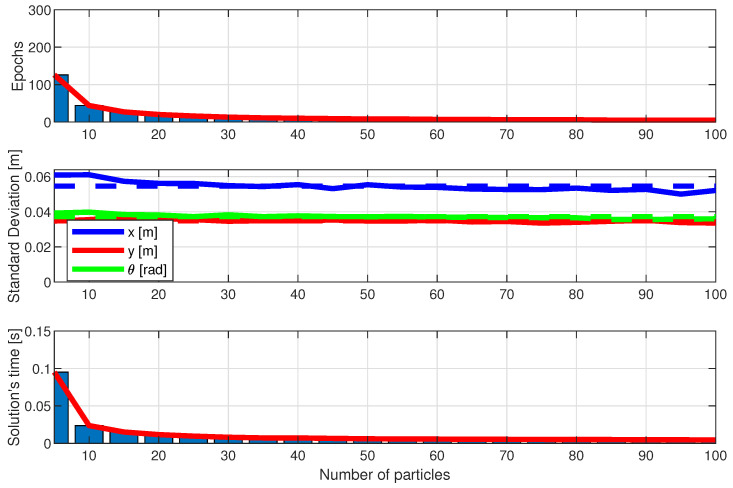
Performance of the MRFO algorithm in terms of the number of particles: convergence speed (in epochs), error standard deviation (in meters), and computational speed to solution (in seconds).

Figure 16, Figure 17 and Figure 18 show the performance of the LBBA proposed here, the MRFO algorithm, and the BWO algorithm. They show the algorithm’s performance considering the three criteria described in the previous subsection, using three plots: the first one shows the convergence speed in epochs, the second one shows the standard deviation of the error between the estimated robot position/orientation and the real ones, and the third one shows the computation time to calculate the robot position, always considering the number of particles as a parameter.

The results show that our LBBA performed better, considering the three criteria. This algorithm demonstrated fast convergence, high computational efficiency, and good final solution quality, proving to be a robust and practical choice for robot localization applications. Specifically, the LBBA stood out for its simplicity of implementation and lower number of computational steps without compromising the final solution’s precision. Notice that the BWO algorithm outperformed the LBBA regarding error standard deviation, but its convergence and the computation time needed to obtain a solution were worse. Regarding the MRFO algorithm, one can see that it performed well in terms of convergence speed and computation time to obtain a solution but showed a more significant error standard deviation than the BWO algorithm and LBBA. Therefore, considering the whole performance, the LBBA proposed here is an excellent tool for robot localization throughout its navigation.

Therefore, the LBBA proposed here stands out as a choice for embedded systems in mobile robotics due to its balance between rapid convergence and sufficient accuracy, achieved with a low computational cost. Although the BWO algorithm demonstrated superior accuracy, it demanded a significantly higher computational time, making it less viable for real-time applications with limited processing power and battery capacity. The MRFO algorithm, on the other hand, achieved faster convergence with fewer particles but at the expense of precision, which is critical for reliable localization. The LBBA proposed here successfully bridges these two extremes, providing an efficient solution that combines suitable accuracy with computational efficiency, thus meeting the stringent requirements of embedded systems, where processing speed and resource optimization are essential.

## 5. Conclusions

This work presents a detailed analysis of the fusion of LIDAR sensor data and a digital compass for the position estimation of a LIMO robot, showing significant gains in efficiency and accuracy. A video of the experiment is available at https://youtu.be/QhTW7dMyVK4 (accessed on 10 January 2025). The main conclusion of this study is that integrating these sensors allowed a significant reduction in the number of particles used in the random search-based localization algorithm (the leader-based bat algorithm (LBBA)), which resulted in a substantial reduction in computation. This optimization significantly improved the time required to obtain the global localization of the robot, making the discussed methodology a viable and effective tool for control experiments that require precise localization in real time.

In addition, the results demonstrated that, although the navigation system maintains a high average accuracy in different trajectories, the geometric complexity of these trajectories can cause considerable error peaks. This behavior suggests the need to improve the localization algorithm, and machine learning techniques are a promising possibility. These techniques could be explored to predict and dynamically adjust localization errors, mainly when accomplishing more complex trajectories. Similarly, adopting advanced adaptive control techniques can mitigate these peaks, improving the performance of autonomous navigation in challenging scenarios.

Another highlight was real-world testing using the C++ programming language, which proved effective in controlling the robot using the position estimation provided by the localization algorithm exclusively. The embedded implementation of these codes in the robots could bring additional processing gains and reduce packet loss in the network. This is a crucial aspect for applications that demand low latency and high reliability in control computer–robot communication.

Incorporating a UAV (drone) with ground vehicles presents an avenue for further exploration in this study. This method would enable the examination of the robot’s localization in three dimensions, extending beyond the XY plane of the known map. Additionally, integrating data from extra location sensors would enhance the accuracy and reliability of the system, particularly in intricate three-dimensional settings.

In conclusion, this study greatly advances localization methods for mobile robots, showing that effective sensor fusion and refined control algorithms are valuable approaches to enhance the performance and efficiency of autonomous navigation in dynamic settings.

## Figures and Tables

**Table 1:** Eliptic Case: Root Mean Square Error (RMSE) comparisons for LBBA and filtered LBBA.

Error Metric	RMSE X (m)	RMSE Y (m)
LBBA	0.0494	0.0238
LBBA Filtered	0.0223	0.0081

**Table 2:** Leminiscate Case: Root Mean Square Error (RMSE) comparisons for LBBA and filtered LBBA.

Error Metric	RMSE X (m)	RMSE Y (m)
LBBA	0.0330	0.0181
LBBA Filtered	0.0056	0.0004

## Data Availability

Data is contained within the article.
